# Frameshift mutations in the *mmpR5* gene can have a bedaquiline-susceptible phenotype by retaining a protein structure and function similar to wild-type *Mycobacterium tuberculosis*

**DOI:** 10.1128/aac.00854-24

**Published:** 2024-10-24

**Authors:** J. Snobre, C. J. Meehan, W. Mulders, L. Rigouts, R. Buyl, B. C. de Jong, A. Van Rie, O. Tzfadia

**Affiliations:** 1 Department of Biomedical Sciences, Institute of Tropical Medicine, Antwerp, Belgium; 2 Department of Biosciences, Nottingham Trent University, Nottingham, United Kingdom; 3 Faculty of Medicine and Health Sciences, University of Antwerp, Antwerp, Belgium; 4 Vrije Universiteit Brussel, Brussels, Belgium; St. George's, University of London, London, United Kingdom

**Keywords:** *Mycobacterium tuberculosis*, drug-resistant tuberculosis, bedaquiline, Rv0678, frameshift

## Abstract

Bedaquiline (BDQ) is crucial for the treatment of rifampicin-resistant tuberculosis, yet resistance threatens its effectiveness, mainly linked to mutations in the *mmpR5* (*Rv0678*) gene. While frameshift mutations are thought to produce non-functional proteins, we hypothesize that they can result in conserved proteins through late-stop codons or alternative reading frames and remain BDQ susceptible. We extracted 512 isolates harboring frameshift mutations in *mmpR5* from the World Health Organization (WHO) catalog and 68 isolates with minimum inhibitory concentration (MIC) in mycobacterial growth indicator tube (MGIT) through a literature review. Using BioPython and AlphaFold2 we computed open (ORF) and alternative reading frames (ARFs) sequences and protein structures and assessed similarity to the wild type using an alignment and template modeling (TM)-score. Among the WHO 512 isolates, 24.8% were BDQ-sensitive. Out of 184 unique frameshift mutations with available nucleotide information, a late-stop codon in the ORF occurred for 32% of the mutations. Also, 40.7% resulted in a conserved sequence, through the ORF or one of the forward ARFs. In 68 isolates with available MGIT MIC data, the presence of late-stop codons in the ORF (OR 4.71, 95% CI 1.36–19.3) or a conserved reading frame (OR 10.4, 95% CI 2.07–102.9) were associated with BDQ sensitivity. Protein structures from the conserved sequences showed high similarity (TM > 0.8). We show that frameshift mutations may retain BDQ susceptibility through late-stop codons in the ORF or conserved ARFs. These findings could improve the prediction of the BDQ phenotype from genomic data and have important implications for treatment decisions. Research Foundation—Flanders, Academy of Medical Sciences, the Wellcome Trust, the Government Department of Business, Energy and Industrial Strategy, the British Heart Foundation and Diabetes UK, and the Global Challenges Research Fund.

**IMPORTANCE:**

Tuberculosis (TB), caused by *Mycobacterium tuberculosis*, remains the deadliest infectious disease and is particularly challenging to treat when it becomes drug-resistant. Bedaquiline (BDQ) is a recently recommended core drug for treating drug-resistant TB. However, resistance to bedaquiline is already emerging, primarily due to mutations in the *mmpR5* gene. Identifying which mutations cause resistance and which do not is a critical knowledge gap. In particular, little is known about the effect of frameshift mutations, typically thought to make TB bacteria resistant to bedaquiline by producing non-functional proteins. Yet, one-quarter of isolates with a frameshift mutation are still susceptible to bedaquiline. How the bacteria produce a functional protein despite the frameshift mutation is unknown. We analyzed over 500 frameshift mutations using computational methods to model their effects on protein structure and bedaquiline resistance. Our findings revealed that some frameshift mutations can still produce functional proteins, allowing bacteria to remain sensitive to bedaquiline. Specifically, bacteria can produce a functional protein despite frameshift mutations if the mutation occurs near the end of the protein or if an alternative reading frame is available. These insights improve our ability to interpret mutations associated with bedaquiline, the most important drug for drug-resistant TB, allowing more accurate and effective treatment decisions.

## INTRODUCTION

The management of rifampicin-resistant tuberculosis (RR-TB) significantly challenges tuberculosis (TB) control programs. Until recently, treating RR-TB involved intricate multidrug regimens associated with severe side effects and low success rates (1). Since 2019, the World Health Organization (WHO) incorporated new and repurposed drugs in its guidelines for treating RR-TB (2, 3). Bedaquiline (BDQ) stands out as one of the most powerful drugs due to its high efficacy and good safety profile (4, 5). Unfortunately, the use of BDQ is associated with increasing resistance (6), and in some cases with poor treatment outcomes (7).

Most clinical BDQ-resistant strains exhibit mutations in the *mmpR5* gene (8)*,* encoding for a repressor protein that regulates the MmpL5-MmpS5 efflux pump. *mmpR5* displays a wide array of mutations, with varying effects on minimum inhibitory concentrations (MICs). A critical knowledge gap is the correlation between mutations and their impact on phenotypic resistance, and the clinical breakpoint MIC. This knowledge is vital for preventing the administration of BDQ-containing regimens to patients harboring BDQ-resistant strains, which could lead to treatment failure, resistance amplification, and transmission of BDQ-resistant Mtb strains. At the same time, excluding BDQ from an RR-TB treatment regimen should only occur when BDQ resistance is diagnosed with confidence, as such a decision may subject individuals to prolonged, less effective, and more toxic RR-TB treatment alternatives.

In recent years, extensive efforts have been dedicated to linking single nucleotide polymorphisms (SNPs) and frameshift mutations in *mmpR5* to BDQ phenotypic resistance. In the WHO catalog of mutations in Mtb, this is done using a statistical approach. Alternative approaches modeling the effect of the mutation in protein structure concentrate solely on SNPs (9). Frameshift mutations, constituting up to 40% of all variants (10), are often inherently assumed to yield non-functional, truncated proteins. In the case of *mmpR5*, a truncated repressor protein resulting from a frameshift mutation would fail to repress the efflux pump, resulting in its overexpression and expulsion of BDQ, leading to high BDQ MIC values. Based on this assumption, the second version of the WHO catalog introduced an expert rule stating that any loss of function mutation in *mmpR5* is associated with BDQ resistance (2). Nevertheless, multiple reports report that isolates carrying frameshift mutations can exhibit a spectrum of MIC values, from sensitive to high resistance (11, 12). This variability challenges the assumption that frameshift mutations inevitably result in resistance.

Most studies investigating the impact of mutations in *mmpR5* have viewed the sequence of the gene as linear. In reality, genes code for proteins that possess a three-dimensional, tertiary folded structure, and it has been shown that, despite differences in sequence, the same genetic information can yield a similar folding, thereby preserving a conserved protein structure and function (13). The crystal structure of *mmpR5* as detailed by Radhakrishnan et al. in 2014 (14), shows a dimeric two-domain molecule with an architecture similar to members of the MarR family of transcriptional regulators. Functional domains identified are the DNA-binding (codons 34-99) and dimerization domains (codons 16-32 and 101-160). Furthermore, studies in Mtb assume that proteins are exclusively translated through a single open reading frame (ORF) originating at nucleotide 1 even though a given amino acid sequence can undergo translation in six different alternative reading frames (ARFs) (three forward and three reverse) in bacteria (15). In *Escherichia coli* and *Pseudomonas aeruginosa*, frameshifted proteins were shown to maintain their functionality through ARFs nested within the ORF (16, 17).

In this study, we hypothesize that frameshift mutations in *mmpR5* that result in a protein structure similar to the wild type through a late-stop codon in the ORF or through conserved ARFs can result in Mtb strains with a BDQ susceptible phenotype. We also examined whether the conservation of functional domains in frameshifted proteins is associated with susceptible BDQ phenotype. Finally, we leverage the artificial intelligence tool AlphaFold2 to investigate the impact of frameshift mutations in *mmpR5* on the protein structure. In this way we provide new insights into the functionality of mutated proteins, challenging the principle that frameshift mutations always yield non-functional proteins. This has important implications for interpreting genotypic test results for BDQ when performed for clinical care.

## MATERIALS AND METHODS

### Data set description

**Fig 1:**
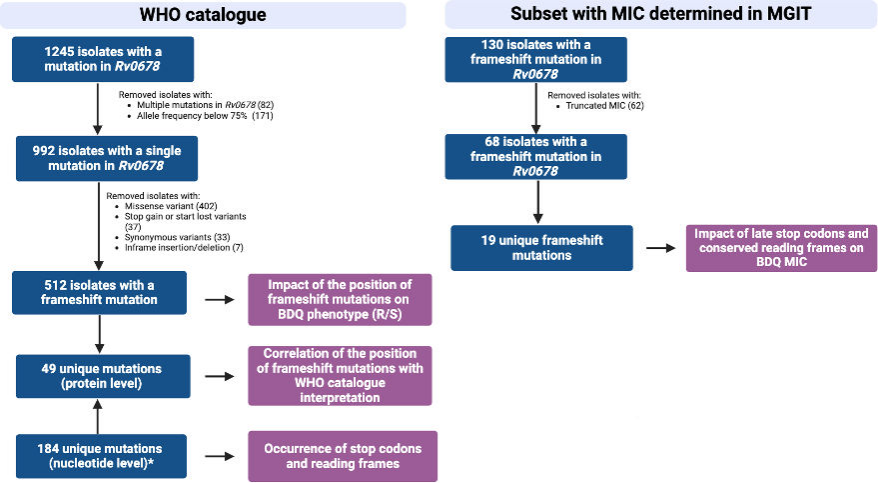
Flowchart of data sets used in this study. *Different mutations at the nucleotide level can result in the same frameshift mutation at the protein level. The WHO data set was extracted from the “Genomic Coordinates” file in the WHO catalog version 2, which includes the reference and alternative nucleotides for each mutation but the corresponding isolate numbers and their respective BDQ phenotypes are not presented.

The analysis included isolates reported in the second edition of the WHO catalog (2). Of 1,245 Mtb isolates exhibiting a mutation in the *mmpR5* gene, we excluded 82 isolates that had multiple mutations in *mmpR5* and 171 with allele frequency below 75% (Fig. 1). Of the remaining 992 isolates, 512 isolates (51.6%) harbored a frameshift mutation. A data set of these 512 isolates and their phenotype categorized as binary resistant/susceptible using diverse phenotypic methods considered acceptable for inclusion in the WHO catalog was used to analyze the impact of the position of frameshift mutations on BDQ phenotype (Fig. 1).

Next, we assessed if the position of 49 unique frameshift mutations in the *mmpR5* gene represented in the 512 isolates was associated with a classification “associated with resistance” (category 1) in the WHO catalog. To assess whether the frameshift mutations in the WHO data set result in late-stop codons in an ORF or ARF, we utilized a collection of 184 isolates with available nucleotide information (Fig. 1).

To assess the impact of late-stop codons, conserved reading frames, and BDQ MIC for each frameshift mutation, we used published clinical isolates (12, 18–26) with a *mmpR5* frameshift mutation for which the phenotype was determined by MGIT (critical concentration 1 µg/mL). Only isolates with a single mutation in *mmpR5* and without mutations reported in *atpE* and *pepQ* were collected as the presence of these additional variants could confound the genetic causality of BDQ resistance. Of the 130 isolates collected, 62 were excluded as they presented a truncated MIC (Fig. 1).

### Statistical testing

We used Fisher’s Exact Test to evaluate the associations between variables of interest and BDQ phenotype. Statistical analyses were conducted using an R script (R version 4.1.2) with the stats package.

### Assessing the presence of late-stop codons and generation of alternative reading frames and assessing alignment scores

An in-house Python script utilized the BioPython package to translate each nucleotide sequence into amino acid sequences (27). This translation was performed according to the bacterial genetic code, considering the three forward reading frames (ORF and two ARFs) and three reverse reading frames. We defined late-stop codons as stops occurring after codon 160, marking the end of the functional domains in the *mmpR5* gene. For each ARF, we determine the similarity to the wild type in terms of percentage sequence alignment. Alignment scores were computed after using the Biopython pairwise2 module for global sequence alignment (28). The cutoff of 85% was used to indicate high sequence similarity (29, 30).

### Generating the AlphaFold2 model and assessing protein structure similarity

To produce a three-dimensional model of the *mmpR5* protein, we utilized ColabFold which leverages advanced deep-learning algorithms to predict protein structures (7). The amino acid sequence of *mmpR5* retrieved from MycoBrowser was input into ColabFold to produce a 3D model of the *mmpR5* protein represented by a Protein Data Bank file which describes the protein’s structural arrangement and respective template modeling score (TM-score) (31) to assess the similarity with the Mtb wild type, with a score greater than 0.5 indicating high structural similarity.

## RESULTS

### Impact of the position of frameshift mutations in the *mmpR5* gene on BDQ phenotype

**Fig 2:**
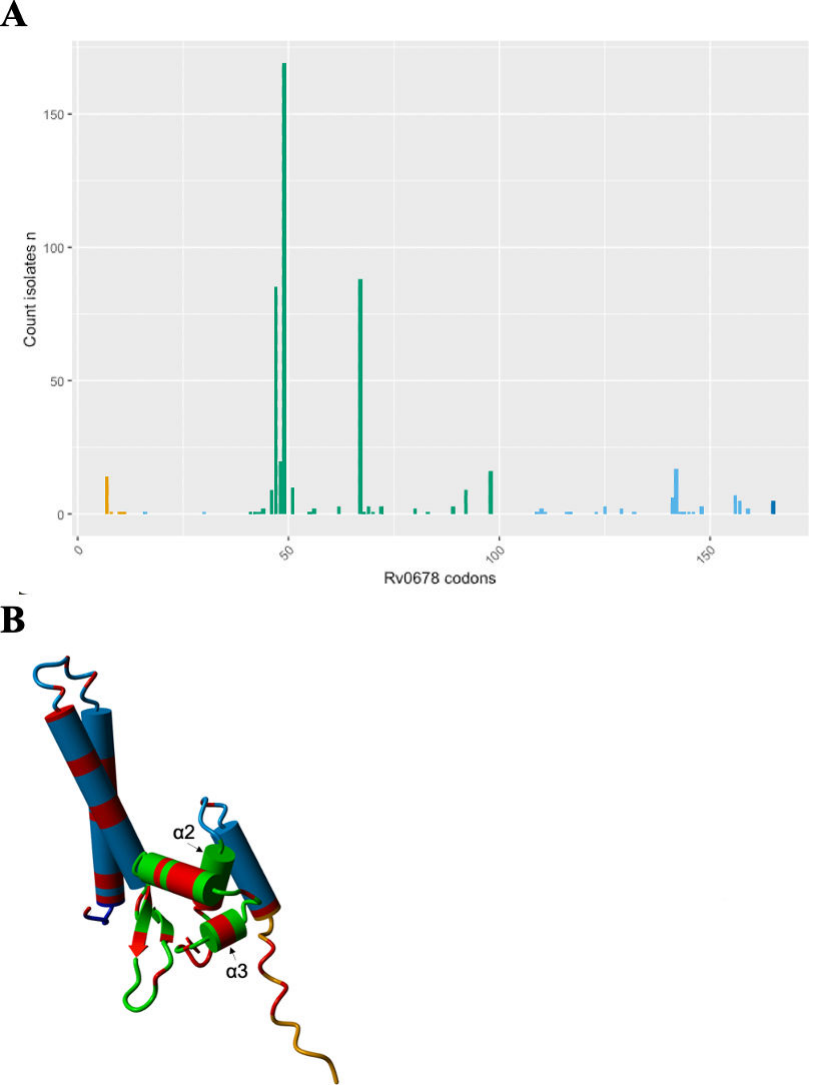
Distribution of frameshift mutations within (A) the mmpR5 gene and (B) the protein structure. The different domains are represented by different colors: orange for the beginning of the protein, light blue for the dimerization domain, green for the DNA-binding domain (including the α2 and α3 helices), and blue for the end of the protein. In the protein structure, the locations of the mutations are marked in red.

Of the 512 isolates with a single frameshift mutation reported in the WHO catalog, 385 (75.2%) were phenotypically BDQ-resistant (R), while 127 (24.8%) were sensitive (S). Among these 512 isolates, most mutations (84.2%, 431/512, of which 324 R, 107 S) occurred in the DNA-binding domain (amino acids 34-99), with more than half (59.4%, 304/512; of which 245 R and 59 S) in the α2-α3 helix (residues 36-62) (Fig. 2). The frameshift variants for the remaining 81 isolates occurred in the dimerization domain (11.5%, 59/512, of which 46 R and 13 S, amino acids 16-33 or 100-160), or were positioned outside the functional domains (4.3%, 22/512, of which 15 R and 7 S) (Fig. 2).

The presence of mutations within the functional domains (combined DNA-binding and dimerization domain), the DNA-binding domain, or the dimerization domain separately, was not associated with BDQ resistance compared to the presence of frameshift mutations outside these respective domains (OR 1.43, 95% CI 0.48–3.85 *P* 0.45; OR 0.99, 95% CI 0.54–1.76 *P* 1; OR 1.18 CI 0.6–2.49, *P* 0.75).

Conversely, the presence of mutations in the α2-α3 helix of the DNA-binding domain was associated with phenotypic BDQ resistance compared to mutations outside the α2-α3 helix (OR = 2.01, 95% CI 1.32–3.09 *P* 0.0008).

The 49 unique frameshift mutations described in the WHO catalog version 2 were distributed throughout the *mmpR5* gene: 22 were situated in the DNA-binding domain, 22 in the dimerization domain, 4 preceding the functional domains, and 1 following the functional domains. Based on statistical analysis, 12 variants were classified by the WHO catalog as “associated with resistance” and 37 as “uncertain significance.” The location in the functional domain (OR 0.45, 95% CI 0.04–6.1 *P* 0.58), DNA-binding domain (OR 2.02, 95% CI 0.45–9.7 *P* 0.33), the α2-3 helix (OR 2.98 CI 0.57–15.3 *P* 0.13), or the dimerization domain (OR 0.32, 95% CI 0.048–1.57 *P* = 0.18) was not associated with a WHO catalog classification as “associated with resistance.”

### Late-stop codons and conserved reading frames

**Fig 3:**
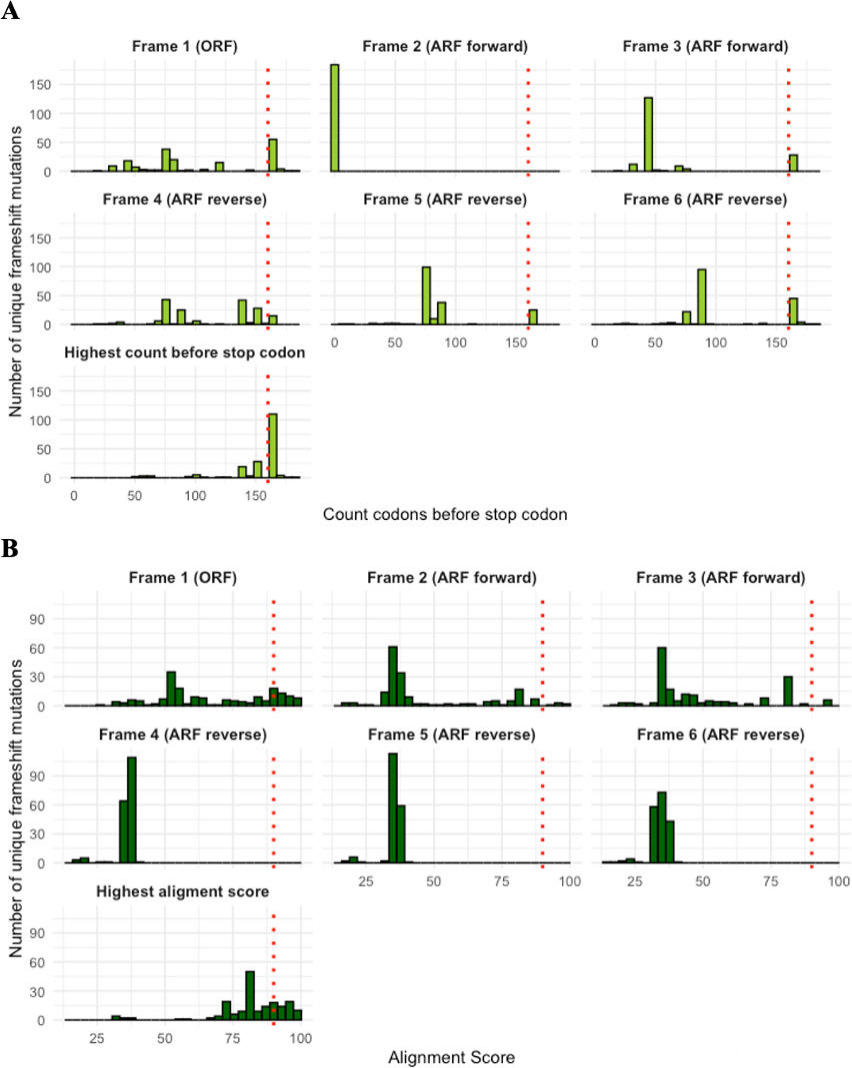
Distribution of (A) the number of generated codons before encountering a stop codon, and (B) alignment scores, across open and alternative reading frames for 184 isolates with a frameshift mutation. The red dotted lines represent in (A) the position of amino acid 160, marking the end of the functional domains in the mmpR5 gene, and in (B) the threshold alignment score of 85%, serving as the cutoff for sequence conservation.

For the 184 unique mutations with available nucleotide information, we computed the ORF and 5 ARFs. We then assessed how many mutations induced a late-stop codon in any of these reading frames. Out of 184 unique mutations 68 (36.9%) didn’t result in a late stop in any of the reading frames, 61 (33,2%) produced a late-stop codon within the ORF, while ARFs would result in a late-stop codon for 119 (64,7%) of 184 mutations: 28 through the third (forward) ARF, 15 through reverse frame 4, 25 through reverse frame 5, and 51 through reverse frame 6 (Fig. 3A). Sixty-four (34.7%) resulted in a late-stop codon through two different reading frames.

Of the 184 frameshift mutations, 40.8% (*n* = 75) resulted in a highly conserved reading frame (alignment score > 85%), 54.9% (*n* = 101) had an alignment score between 50% and 85%, and 4.3% (*n* = 8) an alignment score below 50%. The highest similarity with the wild-type sequence occurred through the ORF (54 out of 184), followed by the second (13 out of 184), or third (8 out of 184) ARF (Fig. 3B). None of the reverse reading frames demonstrated a sequence similar to the wild type. No mutations with a conserved reading frame exhibited high conservation for more than one frame.

### Impact of late-stop codons and conserved reading frames on BDQ MIC

We identified 68 published isolates with frameshift mutations and BDQ phenotype determined in MGIT representing 19 unique mutations (Table 1). These 68 isolate mutations were distributed throughout the entire *mmpR5* gene: 6 were located at the beginning of the protein, 47 within the DNA-binding domain, 13 in the second dimerization domain, and 2 at the end of the protein.

**TABLE 1:** Overview of 19 unique frameshift mutations in the mmpR5 gene identified in 68 isolates, complete with corresponding bedaquiline MIC values measured using the Mycobacterial Growth Indicator Tube (MGIT) system^*a*^

Rv0678 mutation	No. of R isolates	No. of S isolates	aa position	aa position category	Stop codon (ORF)	Codons before stop	Frame 1 alignment	Frame 2 alignment	Frame 3 alignment	Frame 4 alignment	Frame 5 alignment	Frame 6 alignment
16delG	0	3	5	Beginning of the protein (aa 0–16)	First dimerization domain (aa 17-33)	31	35.8	37.6	**96.4**	37.6	34.5	33.9
30delG	0	2	10	Beginning of the protein (aa 0-16)	First dimerization domain (aa 17-33)	31	38.2	37.6	**95.2**	37.6	34.5	33.9
43insA	0	1	14	Beginning of the protein (aa 0-16)	DNA binding domain (aa 34-99)	46	43.0	**93.9**	33.9	37.6	35.2	33.3
138insG	4	0	46	DNA binding domain (aa 34-99)	DNA binding domain (aa 34-99)	46	54.5	**80.6**	34.5	38.2	35.8	32.7
139insG	2	0	46	DNA binding domain (aa 34-99)	DNA binding domain (aa 34-99)	80	55.2	**80.6**	34.5	38.2	35.8	32.7
139insGATC	1	1	46	DNA binding domain (aa 34-99)	DNA binding domain (aa 34-99)	81	56.4	**80.6**	34.5	38.8	35,8	33.3
141insC	1	7	47	DNA binding domain (aa 34-99)	DNA binding domain (aa 34-99)	80	55.8	**80.0**	34.5	38.2	35.8	33.3
144insC	15	3	48	DNA binding domain (aa 34-99)	DNA binding domain (aa 34-99)	80	56.4	**80.0**	34.5	38.8	35.8	32.7
192insG	1	1	64	DNA binding domain (aa 34-99)	DNA binding domain (aa 34-99)	80	61.8	**72.1**	35.2	35.8	37.6	32.7
193delG	0	1	64	DNA binding domain (aa 34-99)	DNA binding domain (aa 34-99)	73	60.6	35.8	**73.3**	35.2	36.4	35.8
198insG	3	0	66	DNA binding domain (aa 34-99)	DNA binding domain (aa 34-99)	80	63.0	**72.7**	35.2	35.8	37.6	32.7
274insA	0	2	91	DNA binding domain (aa 34-99)	Second dimerization domain (aa 100-160)	105	**72.7**	62.4	34.5	35.8	38.2	33.3
289delC	1	4	96	DNA binding domain (aa 34-99)	Second dimerization domain (aa 100-160)	121	**71.5**	35.8	61.8	35.8	35.2	35.8
349insC	1	0	116	Second dimerization domain (aa 100-160)	Second dimerization domain (aa 100-160)	118	**81.2**	52.1	35.2	35.2	37.0	33.3
359insG	0	2	120	Second dimerization domain (aa 100-160)	End of the protein (aa 161-165)	166	**82.4**	50.9	35.2	35.2	36.4	32.7
379delG	1	0	126	Second dimerization domain (aa 100-160)	End of the protein (aa 161-165)	165	**83.6**	35.8	50.9	36.4	33.9	35.2
418insG	0	5	139	Second dimerization domain (aa 100-160)	End of the protein (aa 161-165)	166	**89.1**	44.2	35.2	35.2	37.0	34.5
435delT	0	4	145	Second dimerization domain (aa 100-160)	Second dimerization domain (aa 100-160)	144	**90.9**	34.5	43.6	37.0	33.9	36.4
493insG	2	0	164	End of the protein (aa 161-165)	End of the protein (aa 161-165)	166	**99.4**	33.9	37.6	34.5	38.2	34.5
												

^*a*^ The critical concentration for BDQ MIC in MGIT is 1. Information detailed in the table includes the codon affected by the mutation, the gene domain where the mutation is located, the position of the and domain of the resulting stop codon (frameshift mutations in the gene sequence can induce stop codons further down the sequence), and the alignment scores associated with each mutation for open (frame 1) and alternative reading frames (frames 2, 3, 4, 5, and 6). Abbreviations: AA, amino acid; BDQ, bedaquiline; MGIT, Mycobacterial Growth Indicator Tube; ORF, open reading frame. Highest alignment score for each mutation is indicated in bold.

**Fig 4:**
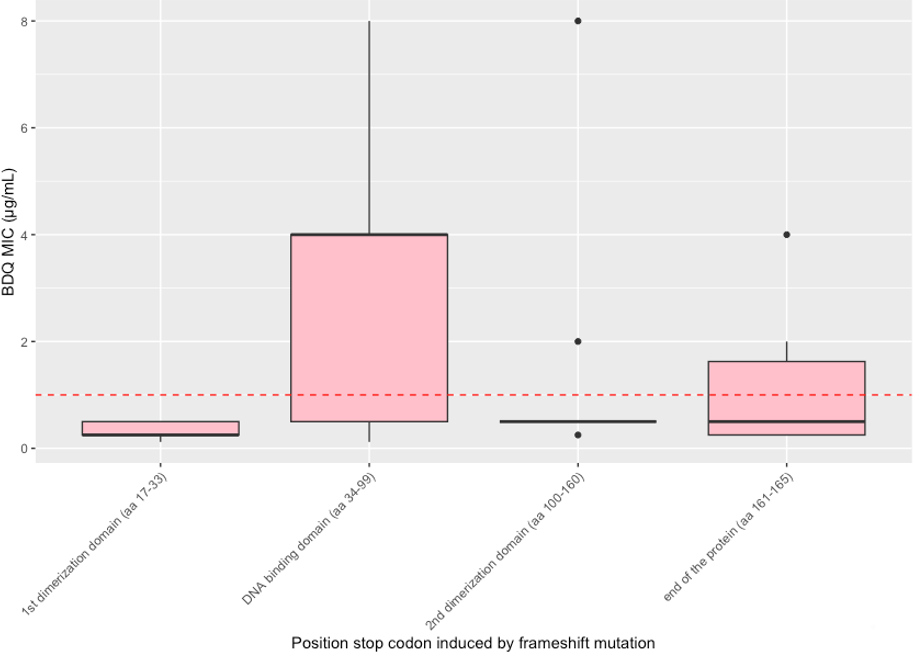
Box plot showing the BDQ-MIC values in MGIT stratified per position of the stop codon in the open reading frame induced by frameshift mutations grouped in domains of mmpR5. Five isolates [5 susceptible (S), 0 resistant (R)] had mutations inducing a stop codon in the first dimerization domain, 43 isolates (16 S, 27 R) in the DNA-binding domain, 10 isolates (8 S, 2 R) in the second dimerization domain, and 10 isolates (7 S, 3 R) at the end of the protein.

First, we explored the impact of stop codons in the ORF induced by frameshift mutations on the BDQ phenotype. Five isolates had mutations inducing a stop codon in the first dimerization domain, 43 isolates in the DNA-binding domain, 12 isolates in the second dimerization domain, and 10 isolates at the end of the protein (Fig. 4). Isolates with stop codons in the first dimerization domain had a very high odds of BDQ susceptible phenotype (OR Infinity) but the 95% CI interval was wide and crossed 1 (0.86—Infinity, *P* 0.055). Isolates with a stop codon in the DNA-binding domain were associated with a resistant BDQ phenotype (MIC > 1) (OR 8.19, CI 2.37–33.9, *P* 0.0002). Isolates with a stop codon in the second dimerization domain (OR 5.63, CI 1.06–57.5, *P* 0.026) were associated with a susceptible BDQ phenotype. Isolates with a stop codon at the end of the protein (OR 2.31, CI 0.47–15.2, *P* 0.31) were not associated with a susceptible BDQ phenotype. Isolates with a stop codon falling after the DNA binding domain (in the second dimerization or at the end of the protein) were associated with a susceptible BDQ phenotype (MIC ≤ 1) (OR 4.71, 95% CI 1.36–19.3, *P* 0.0087).

**Fig 5:**
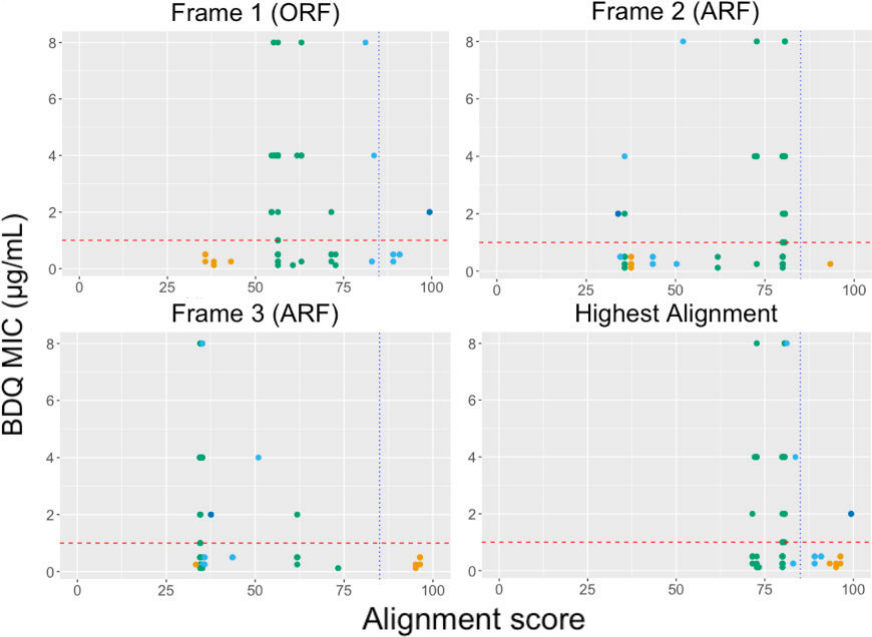
The scatter plot illustrates the correlation between alignment scores and BDQ MIC determined in MGIT across the open reading frame (frame 1) and alternative reading frames (frames 2 and 3) and the frame presenting the highest alignment score for each frameshift mutation. Reverse reading frames are not shown as none of the mutations demonstrated conserved sequences in these frames. Data points are color-coded to represent their locations within the gene’s domains: mutations found at the beginning of the protein before the onset of the functional domains are marked in red; those within the DNA-binding domain are shown in green; and mutations located in the second dimerization domain or at the protein’s terminus are denoted in blue and purple, respectively. The dotted blue line represents the alignment score threshold of 85, while the red dotted line indicates the critical concentration of BDQ MIC at 1.

Next, we explored the impact of conserved ARFs on BDQ phenotype (Fig. 5). The six isolates with frameshift mutations at the beginning of the protein (i.e., a mutation in amino acid position 0-16) showed low alignment scores in the ORF (≤43), yet they achieved a high alignment score (≥94) through a forward ARF (Table 1). The 47 isolates with mutations in the DNA binding domain also had low alignment scores in the ORF (range 54.5 to 72.7), 39 (83%) had a higher alignment score through the second alternative forward reading frame but none achieved a sequence alignment score >80.6%. The 13 isolates with mutations located in the second dimerization domain showed the highest alignment scores through the ORF, ranging from 81.2% to 90.9%, with ARFs having a lower alignment score compared to the ORF. The two isolates with a mutation located at the end of the protein also had the highest alignment scores (99.4) through the ORF. The alignment scores for the reverse reading frames were consistently low (32.7 to 38.2).

Overall, 17 of 68 isolates with a frameshift mutation presented a conserved reading frame (alignment score ≥ 85) in either the ORF or one of the ARFs, while 51 isolates lacked any conserved reading frame. Fifteen of 17 mutations with a conserved sequence occurred in isolates with a BDQ MIC ≤ 1. Thirty out of 51 mutations without a conserved reading frame showed a resistant BDQ phenotype (BDQ MIC > 1 µg/mL). A high alignment score (≥85) for one or more reading frames was associated with a 10.4-fold increase in the odds of a BDQ susceptible phenotype (BDQ MIC < 1 µg/mL) (OR 10.4, 95% CI 2.07–102.9 *P* 0.0007).

**Fig 6:**
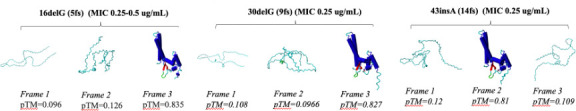
Predicted protein structures. The frameshift mutations situated prior to the functional domains' [16delG (5fs), 30delG (9fs), 43 insA (14fs)] represented in six isolates with low mean BDQ MIC values (<1 µg/mL) and encoded for disrupted open reading frames. However, they led to conserved protein structures through alternative reading frames 2 and 3, similar to the wild type, with high TM scores (>0.8).

The six isolates with frameshift mutations at the beginning of the protein all presented a BDQ MIC < 1. The three unique mutations represented in these isolates were 16delG (5fs), 30delG (9fs), and 43insA (14fs) (Table 1). The alignment scores for these isolates in the ORF were low (≤43) and the ORF led to protein structures that were not conserved (TM scores ≤ 0.12). For each of these three mutations, one ARF had a highly conserved sequence (alignment score ≥ 94). Mutations 16delG (5fs) and 29delG (9fs) exhibited a highly conserved protein structure through ARF 3 (TM-score ≥ 0.83), mutation 43insA (14fs) presented a highly conserved protein structure in frame 2 (TM-score 0.81) (Fig. 6).

## DISCUSSION

In this study, we showed that the varying effects of frameshift mutations in the *mmpR5* gene on the BDQ phenotype can be partly contingent on their effect on the protein sequence. When frameshift mutations induce a late-stop codon, the ORF can still allow structural integrity. Isolates with frameshift mutations can also encode proteins closely resembling the wild type through ARFs resulting in a low BDQ MIC < 1. We observed that a high proportion of the frameshift mutations reported in the WHO catalog result in a late-stop codon (32%) and conserved gene sequences through ARFs (40.2%). In isolates with BDQ MGIT MIC data, we showed that isolates with a late-stop codon in the ORF falling after the DNA binding domain (in the second dimerization domain or after the end of the functional domain) had higher odds of a BDQ susceptible phenotype (OR 4.71, 95% CI 1.36–19.3, *P* 0.0087).

Isolates with conserved sequences through ORF or ARFs had more than 10-fold higher odds of BDQ susceptible phenotype (OR 10.4, 95% CI 2.07–102.9 *P* 0.0007). Our results suggest that incorporating information on the effect of the location of the stop codon in the ORF or the presence of ARFs with high alignment to wild-type Mtb could improve the prediction of the BDQ phenotype from genomic data.

Our results confirm findings in other bacteria that frameshift mutations can result in the production of functional proteins. For *P. aeruginosas*, the translation of reverse reading frames was confirmed by ribosome profiling and mass spectrometry (17). Similarly, ribosome profiling revealed out-of-frame internal minimal reading frames in 13 *E. coli* genes (32). To date, two studies have focused on the functionality of frameshift mutations in *Mtb*. One study found that frameshift mutations in the essential *rpoB* gene (531insC) were not compatible with *Mtb* viability. Surprisingly, this mutation still led to the production of a fully functional rpoB protein (33). In another study, ribosomal profiling revealed widespread translation of non-canonical transcripts, whereby “canonical transcript,” referred to the longest possible protein encoded by the ORF (2).

In the second edition of the WHO catalog of mutations in Mtb (2), it has been introduced an expert rule stating that all frameshift mutations in *mmpR5* that are not statistically associated with resistance are categorized as “associated with resistance interim.” Consequently, any isolate with a loss of function mutation in *mmpR5* is classified as BDQ-resistant. This may lead clinicians to stop BDQ from the RR-TB treatment regimen even when the isolate is still susceptible to BDQ. Our results show that isolates with a frameshift mutation that confers a highly conserved reading frame had 11-fold odds of susceptible BDQ phenotypic. This suggests that the observed variability in the phenotype of isolates with a frameshift mutation in *mmpR5* may not be the consequence of inaccurate phenotypic drug susceptibility test results but of fundamental biological phenomena. Further analyses are needed to assess the impact of frameshift mutations on other drug-resistance-associated genes where the expert rule on loss-of-function mutations is applied, such as those for clofazimine and pyrazinamide.

While associations were observed, the presence of a late-stop codon in the ORF or a conserved ARF did not explain the presence of BDQ susceptibility in all reported instances. In the isolates with available MGIT MIC data, the current reference standard for phenotypic DST, 21 out of 36 isolates with a frameshift mutation with BDQ MIC < 1 did not have a conserved sequence in either the ORF or ARFs. This indicates that other factors allow Mtb to maintain susceptibility to BDQ. One other mechanism could be epistatic interactions involving mutations in the mmpL5/mmpS5 efflux pump, which have been shown to confer hypersensitivity to BDQ (34). Unfortunately, information on mutations in *mmpl5* was available only for nine of the 68 isolates with MGIT MIC information. To better understand the mechanisms of BDQ susceptibility in the presence of frameshift mutations, future publications should provide open access to paired phenotypic and full genome data.

In a data set of 512 isolates with a frameshift mutation extracted from the WHO catalog, we observed an association between mutations in the α2-α3 helix of the DNA-binding domain and phenotypic resistance (OR 2.01, 95% CI 1.32–3.09, *P* = 0.0008) compared to mutations outside the α2-α3 helix. However, of the 304 isolates reported in the α2-α3 helix, 169 harbored the same mutation, Glu49fs. Since we didn’t have access to epidemiological information, we cannot verify whether these isolates belong to the same clone. For this reason, we recommend caution in considering isolates with a mutation in the α2 and α3 helix regions as resistant.

We acknowledge several limitations in our study. The data set with nucleotide information necessary for computing stop codons and reading frames provided as supplementary information to the WHO catalog version 2 is not linked with phenotypic data. Consequently, the association between BDQ MIC and the presence of late-stop codons in the ORF or conserved ARFs was limited to 68 published isolates. Future studies should use larger datasets and perform experiments using CRISPR technologies and mass spectrometry to confirm whether the ARFs with high alignment scores translate to transcription and protein expression that support a susceptible phenotype.

In summary, our findings challenge the principle that all frameshift mutations in *mmpR5* result in a BDQ-resistant phenotype. They suggest that frameshift mutations can preserve protein structure closely resembling the mmpR5 wild type when the stop codon emerges late in the protein sequence or when there is a conserved ARF. A better understanding of the biological processes that explain the presence of a susceptible BDQ phenotype in the presence of a frameshift mutation in *mmpR5* could improve evidence-based treatment decision-making.

## Data Availability

All data are publicly available; references are detailed in the supplemental file. All scripts used for analysis are available in a GitHub repository at https://github.com/jihadsnobre/frameshiftrv0678.

## SUPPLEMENTAL MATERIAL

Supplemental tables aac.00854-24-s0001.xlsx Isolates with a frameshift mutation used for the analysis described in this paper.

ASM does not own the copyrights to Supplemental Material that may be linked to, or accessed through, an article. The authors have granted ASM a non-exclusive, world-wide license to publish the Supplemental Material files. Please contact the corresponding author directly for reuse.
